# Supplementary material for: Mekong delta much lower than previously assumed in sea-level rise impact assessments
Source: Nat Commun. 2019 Aug 28;10:3847. doi: 10.1038/s41467-019-11602-1 (PMC6713785; doi:10.1038/s41467-019-11602-1)
Supplement: Supplementary file 1 — Supplementary Information [file 41467_2019_11602_MOESM1_ESM.pdf]

Supplementary Information for

Mekong delta much lower than previously assumed in sea-level rise impact assessments

Minderhoud et al.

Supplementary Figures

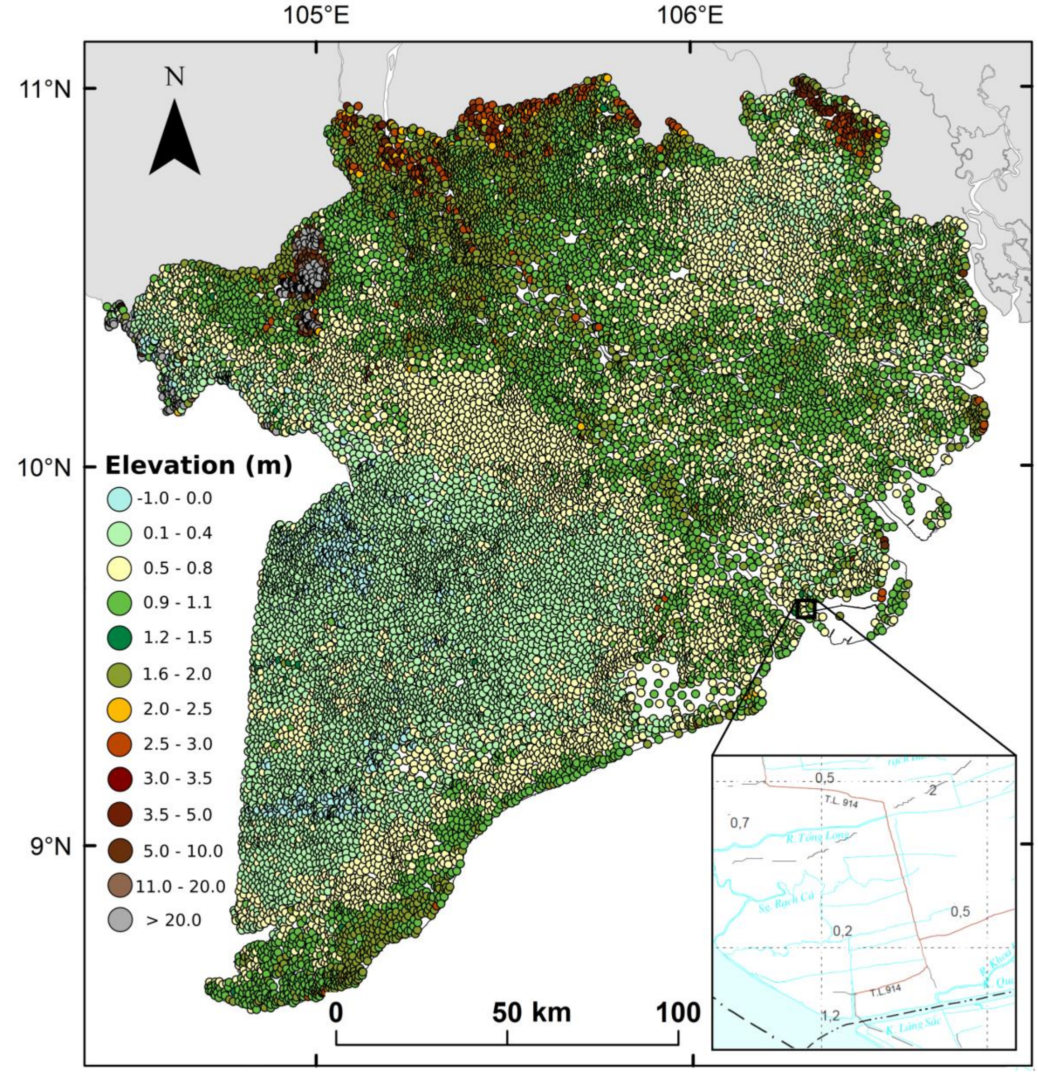

Supplementary Figure 1 | Elevation points used to create the topographical (Topo) DEM. The dataset contains almost 20.000 points derived from a national digital topographical map by the Department of Survey and Mapping of Vietnam (2014).

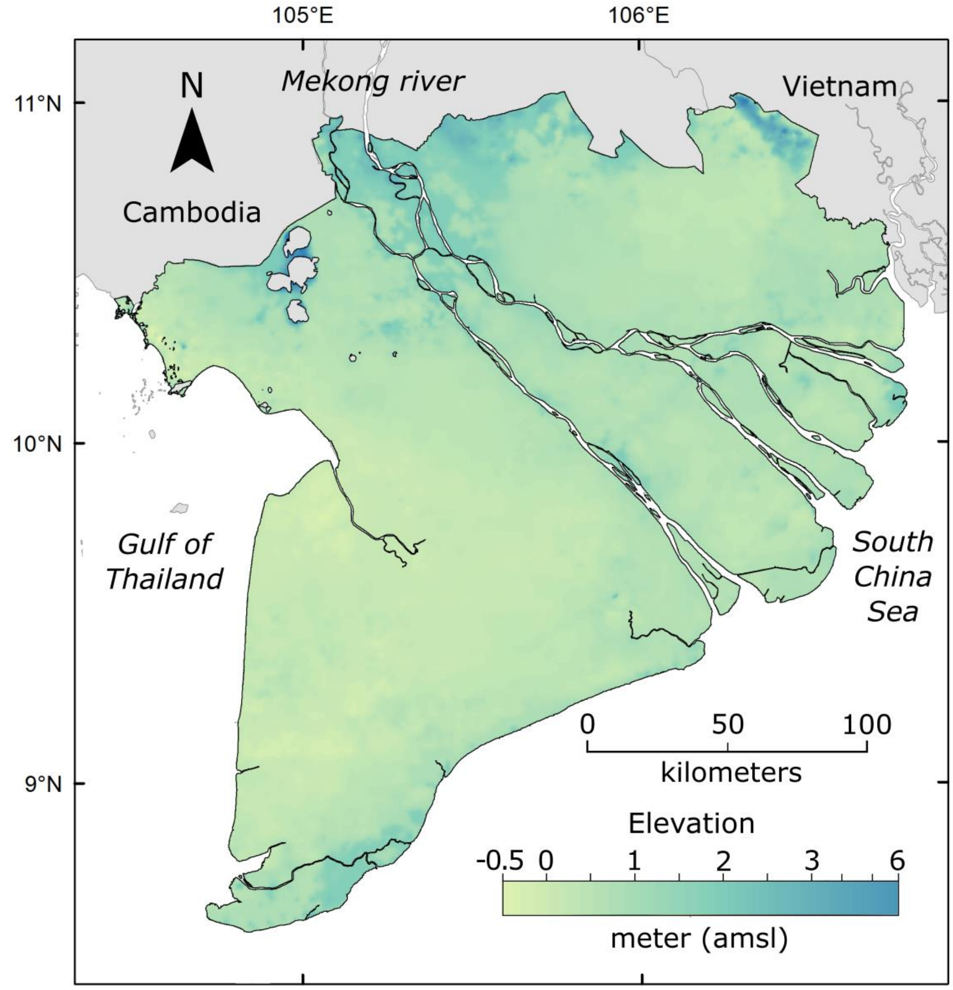

Supplementary Figure 2 | Topo Digital Elevation Model. Similar to Figure 2 in the main manuscript with alternative colour legend to accommodate for colour-blind readers.

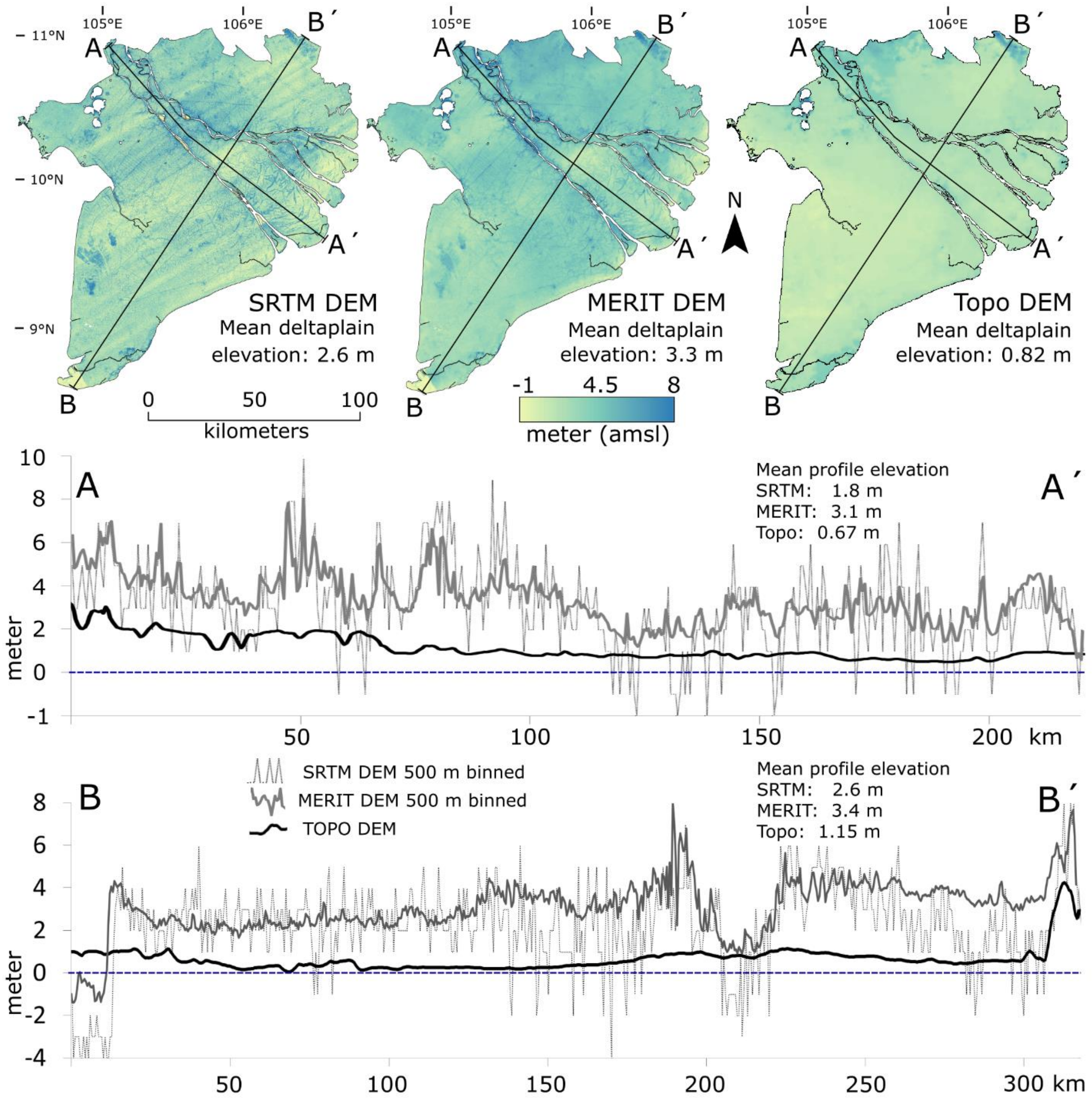

Supplementary Figure 3 | Digital elevation models of the Mekong delta with elevation profiles. Similar to Figure 3 in the main manuscript with alternative colour legend to accommodate for colour-blind readers.

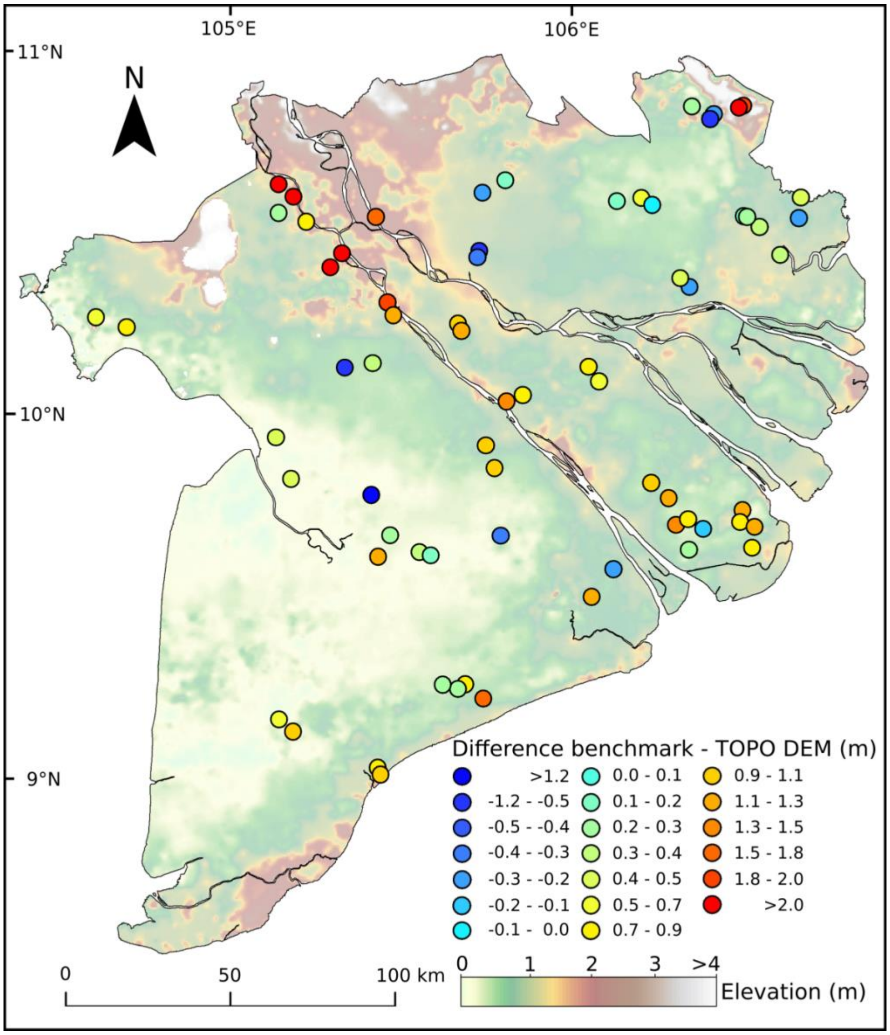

Supplementary Figure 4 | Deviation between independent elevation benchmarks and Topo DEM. Positive values indicate that the benchmark elevation is larger than the Topo DEM elevation.

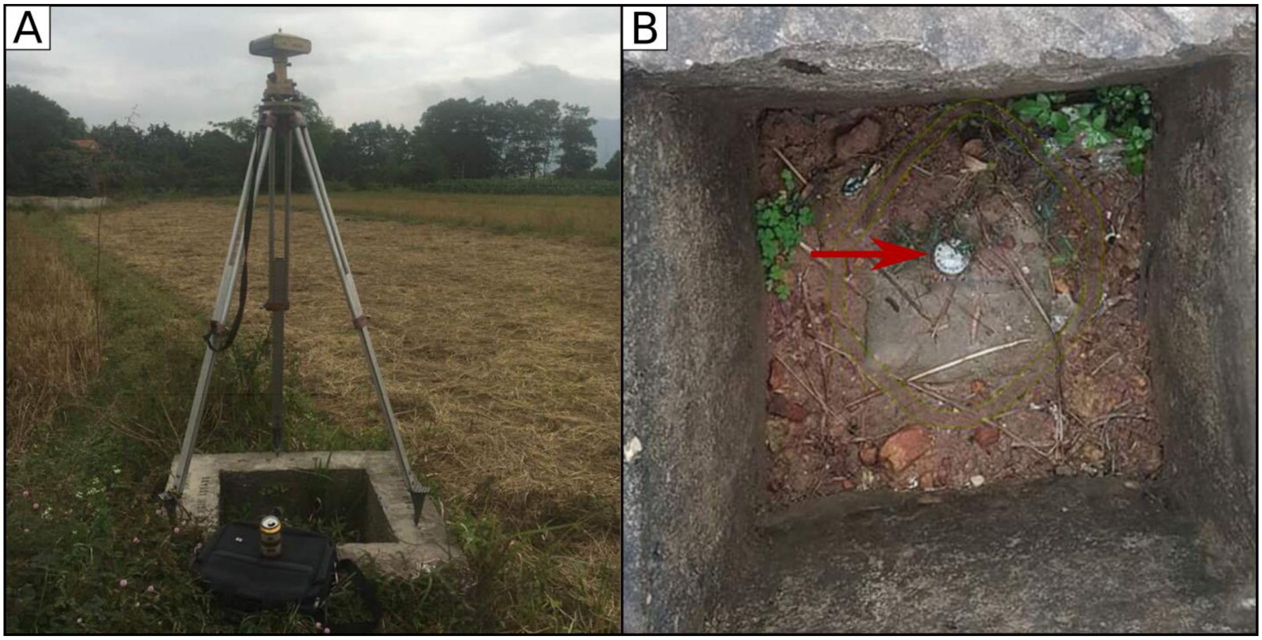

Supplementary Figure 5 | Photographs of national benchmarks in Vietnam. A) Leveling survey in practice to measure the position of a benchmark showing the concrete structure protecting the benchmark. B) Benchmark marker. The markers are installed reportedly 30 cm below the surrounding surface elevation and a larger concrete structure is constructed around the marker for protection.

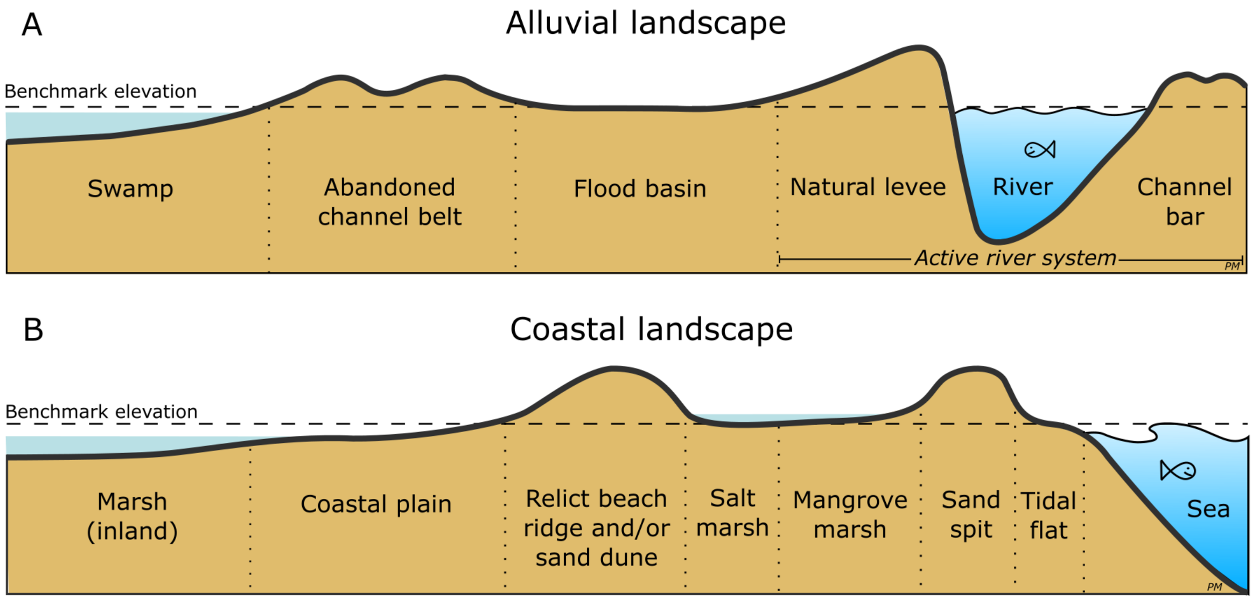

Supplementary Figure 6 | Schematic profiles with geomorphological units of the alluvial and coastal landscapes in the Mekong delta showing their relative elevation. The alluvial landscape (A) is representative for the Upper delta plain and the coastal landscape (B) for the Lower delta plain of the delta. The dashed horizontal line represents a benchmark elevation of the flood basin and the tidal flat for the relative elevation comparison of each geomorphological unit.

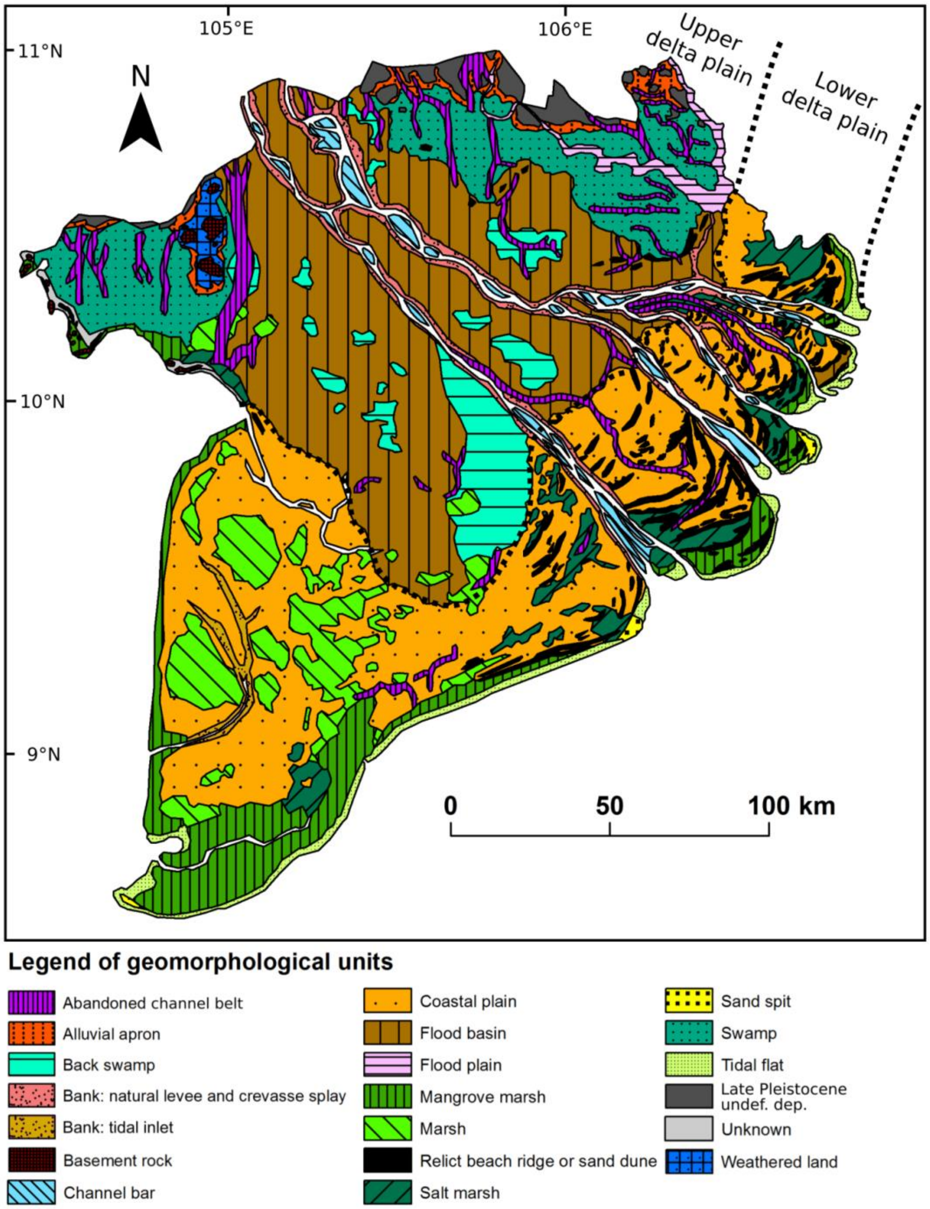

Supplementary Figure 7 | Geomorphological map of the Vietnamese Mekong delta redrawn from Nguyen et al. 2000. The upper and lower Holocene delta plain delineate the separation between the predominantly alluvial and coastal depositional environment.

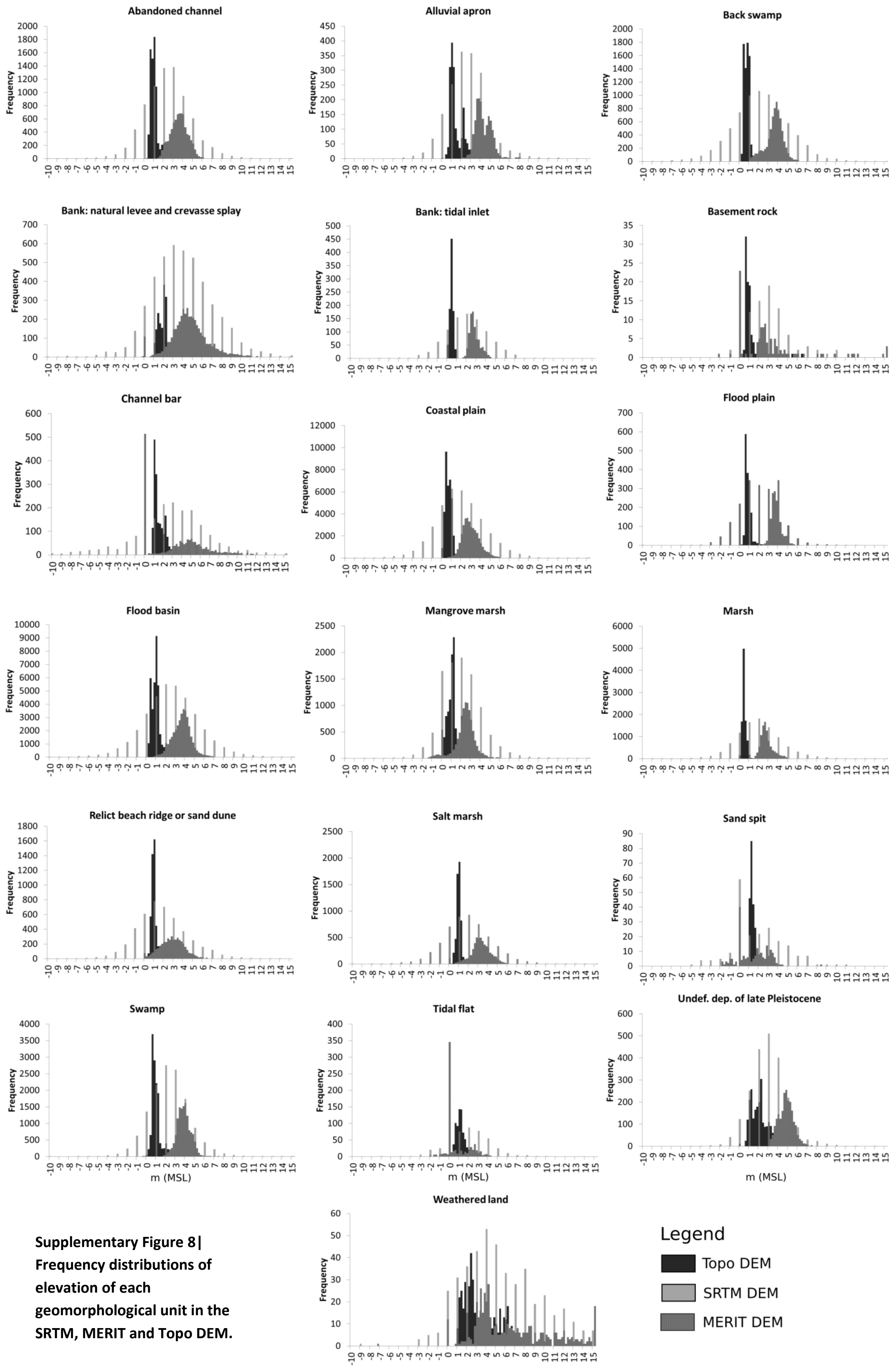

Supplementary Figure 8| Frequency distributions of elevation of each geomorphological unit in the SRTM, MERIT and Topo DEM.

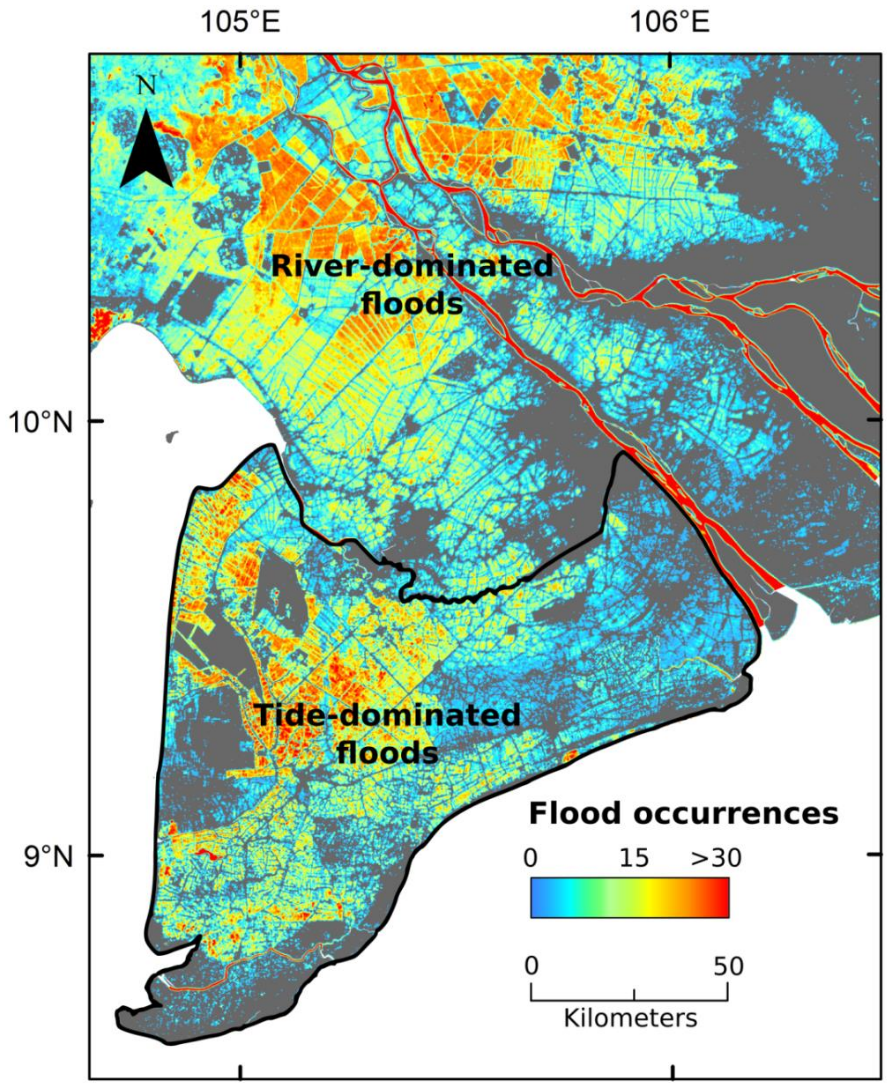

Supplementary Figure 9 | Inundation occurrences in the Mekong delta in the period 2007-2011 derived from ENVISAT ASAR WSM data (Kuenzer et al. (2013). Floods in the upstream part of the Mekong delta are river-dominated and in the southern part tide-dominated. The black line demarcates the tide-dominated flood area used to compare inundation occurrences to elevation of the digital elevation models.

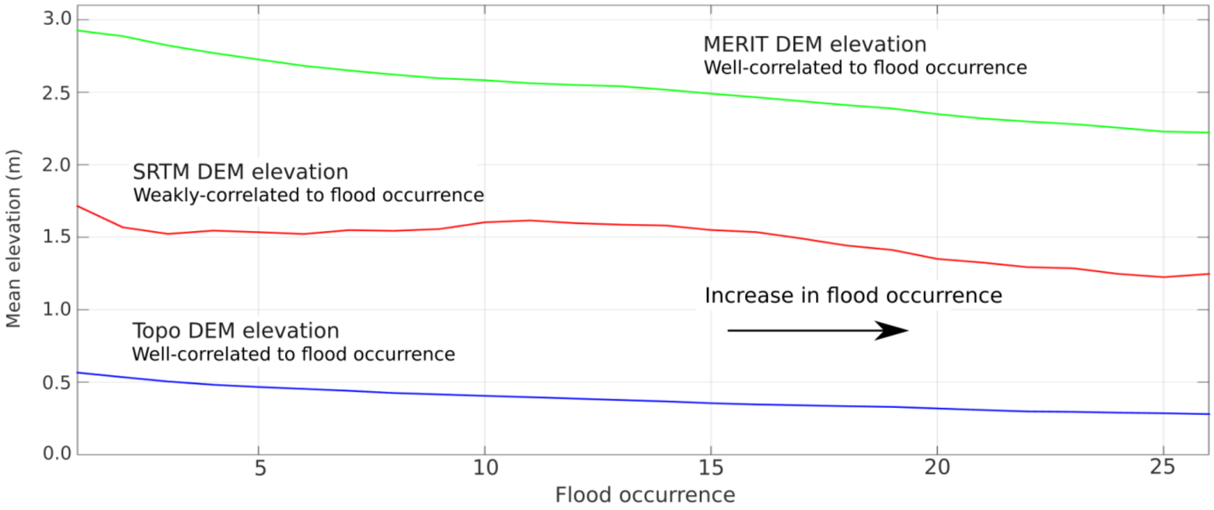

Supplementary Figure 10 | Mean MERIT, SRTM and Topo DEM elevation arranged according to increasing tide-dominated flood occurrence in the southwestern part of the Mekong delta. Flood occurrences were derived from satellite imagery for the period 2007-2011 (Supplementary Figure 9).

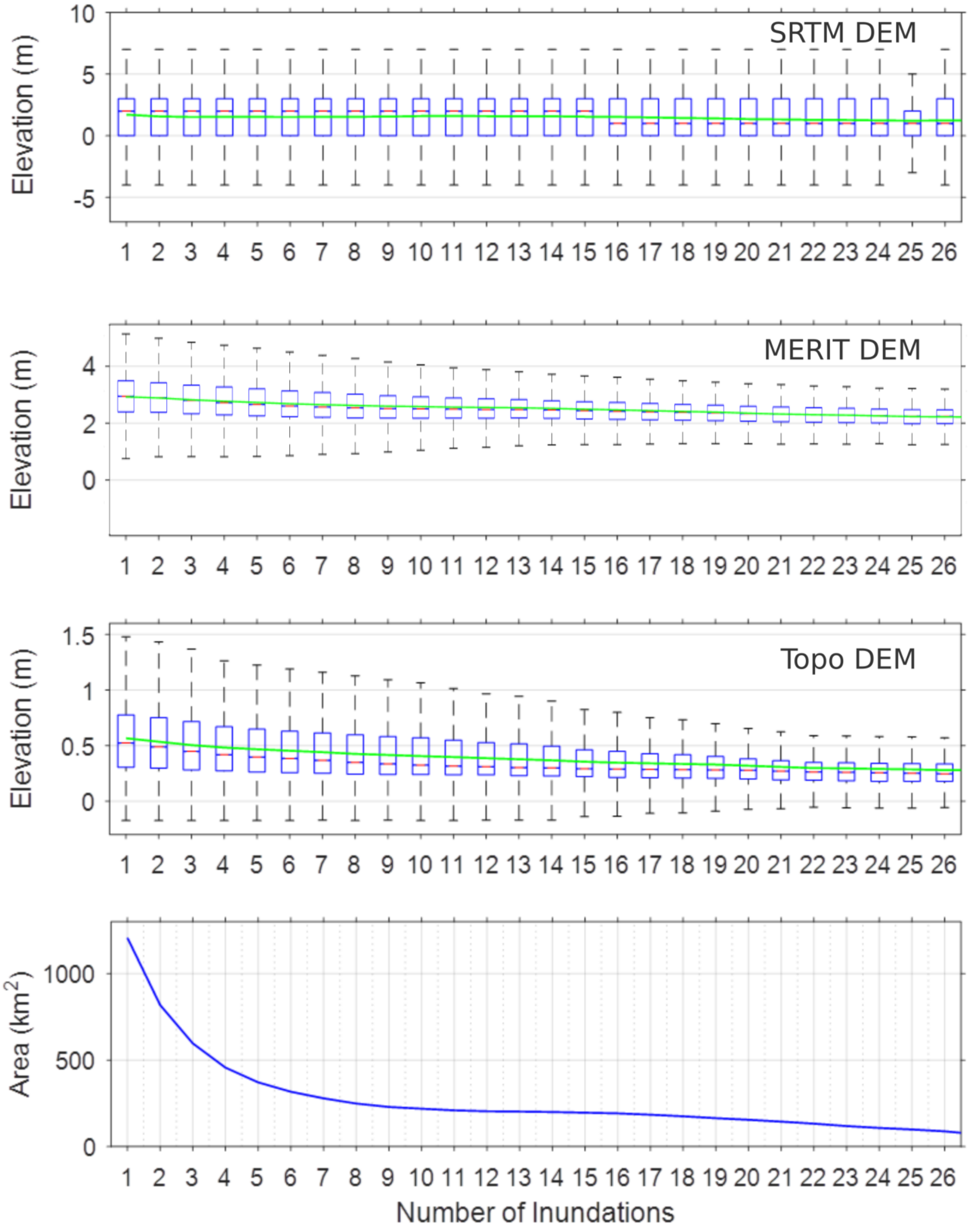

Supplementary Figure 11 | Boxplots showing elevation of the SRTM, MERIT and Topo DEMs for areas with different inundation occurrences in the coastal zone of the Mekong delta. Blue box indicates 25 and 75 percentile, red lines the median, black whiskers the maximum within the 1.5x interquartile range (IQR), green line the average. Extremes are not displayed. Inundation occurrences were derived from 128 satellite images by Kuenzer et al. (2013) between 2007 and 2013 (Supplementary Figure 9).

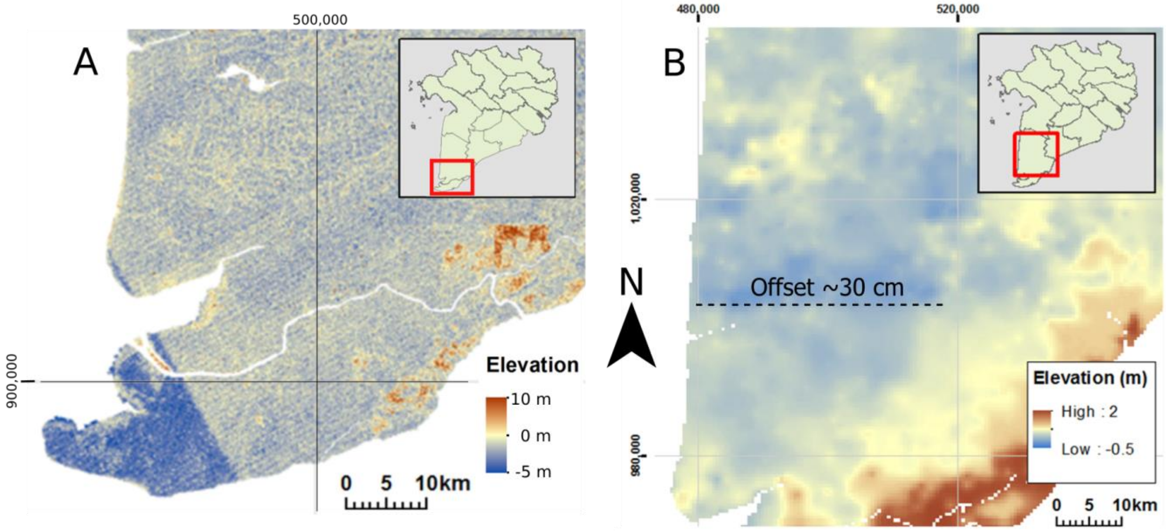

Supplementary Figure 12 | Elevation anomalies present in the SRTM and the Topo DEM. A) In the southwest corner of Ca Mau a sharp and unrealistic elevation change of multiple meters is visible. B) An abrupt elevation change of about ~30 cm is visible along a horizontal line in the Topo DEM.

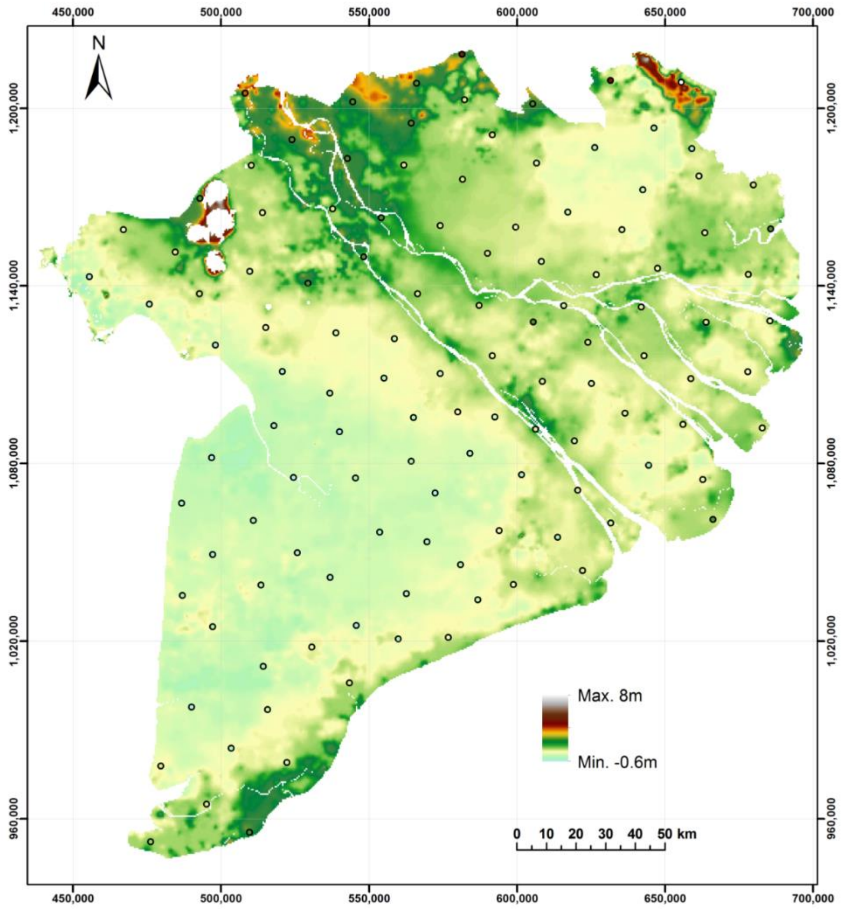

Supplementary Figure 13 | Randomly selected control points used for interpolations validation of the different interpolated DEMs. Colorbar is linear scaled.

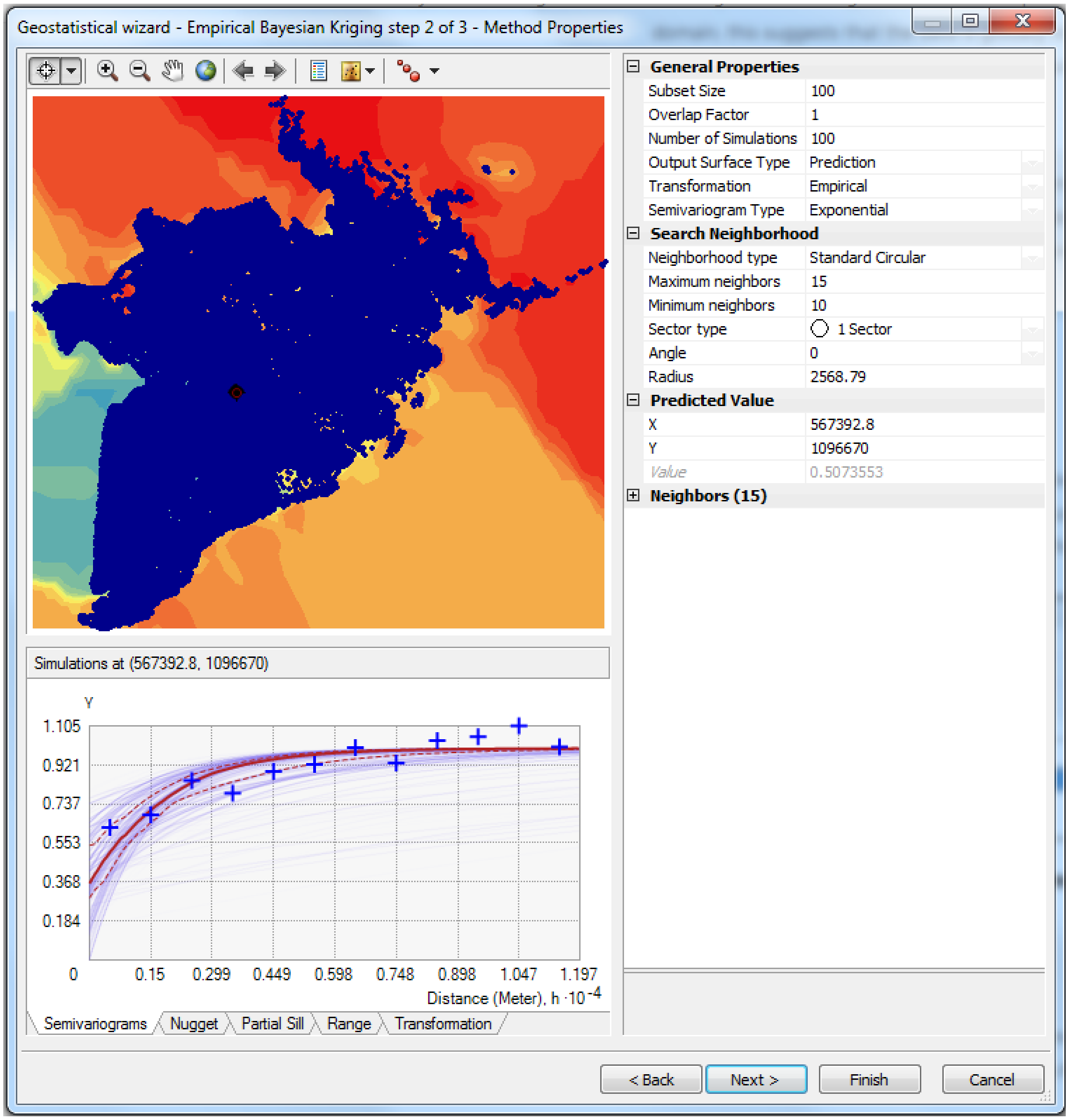

Supplementary Figure 14 | Interpolations settings used to interpolate the elevation points to the Topo DEM. The elevation points were interpolated using empirical bayesian kriging with an empirical data transformation and exponential variogram (ArcGIS version 10.3.1, geostatistical wizard).

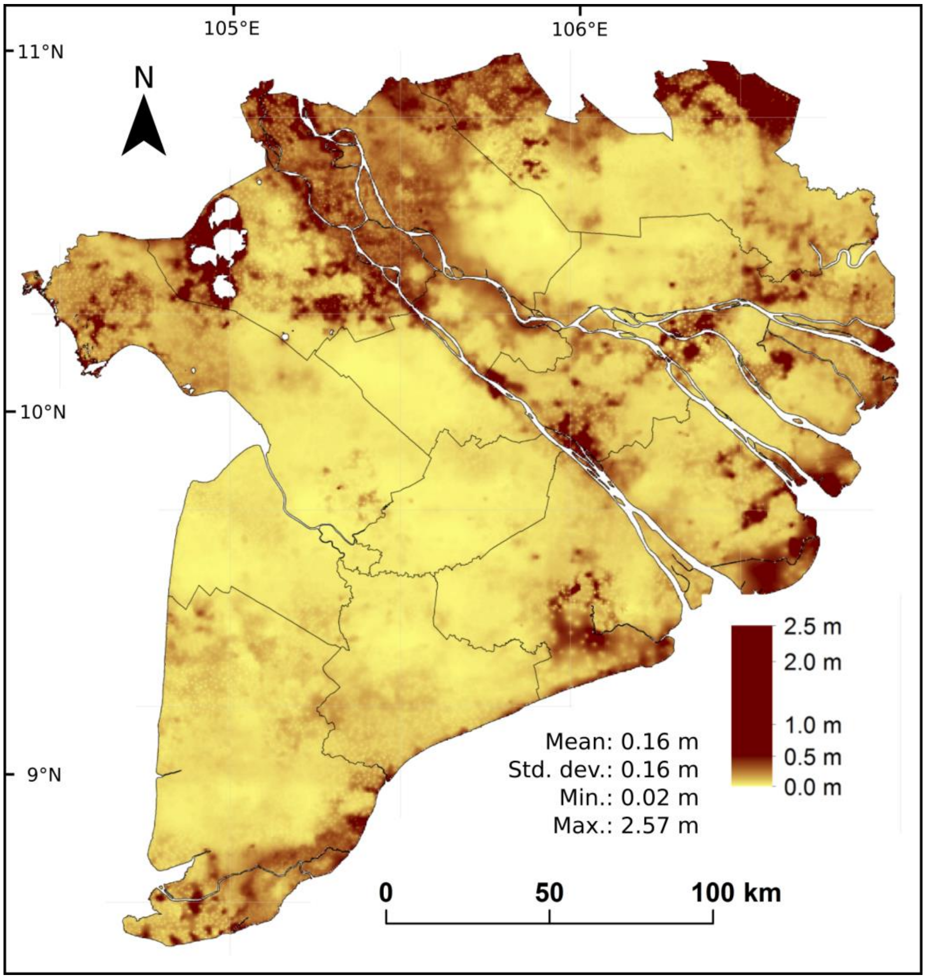

Supplementary Figure 15 | Root mean square error (RMSE) map of the interpolated Topo DEM. Mean RMSE and standard deviation are both 0.16 m. The RMSE increases with decreasing point density (Supplementary Figure 1).

Supplementary Tables

Supplementary Table 1 | Basic statistics of delta plain elevation. Elevation for each province and the entire Mekong delta for the TOPO, SRTM and MERIT DEM. Bedrock outcrops, offshore islands and rivers are excluded.

	Topo DEM				SRTM DEM				MERIT DEM			
Province	Mean (m)	Min. (m)	Max. (m)	SD (m)	Mean (m)	Min. (m)	Max. (m)	SD (m)	Mean (m)	Min. (m)	Max. (m)	SD (m)
An Giang	1.42	0.5	7.8	0.8	3.3	-51	88	3.4	3,8	-13,0	84,1	2,1
Bạc Liêu	0.50	0.1	2.0	0.3	2.0	-21	28	2.3	2,9	-3,6	9,6	0,6
Bến Tre	0.95	0.5	2.6	0.3	2.6	-59	63	2.8	3,1	-7,5	39,5	1,4
Cà Mau	0.59	-0.2	2.6	0.5	2.3	-25	71	2.4	2,4	-11,4	15,8	0,9
Cần Thơ	0.72	0.4	1.8	0.2	2.9	-56	54	3.4	3,9	-23,8	15,2	1,2
Đồng Tháp	1.41	0.7	4.0	0.6	3.7	-34	84	3.0	4,4	-9,2	24,5	1,3
Hậu Giang	0.38	0.1	1.0	0.2	1.9	-82	53	3.2	3,4	-12,6	12,9	0,7
Kiên Giang	0.39	-0.5	5.1	0.3	2.5	-60	107	2.6	3,1	-14,6	57,0	1,0
Long An	1.07	0.2	7.2	0.7	2.4	-46	51	2.3	3,9	-18,0	23,5	0,9
Sóc Trăng	0.68	0.2	2.5	0.3	2.0	-42	42	3.1	3,5	-7,2	17,5	1,0
Tiền Giang	0.85	0.3	1.9	0.2	2.8	-44	40	2.2	3,6	-3,9	14,1	1,0
Trà Vinh	0.79	0.3	2.7	0.2	2.5	-29	37	2.6	2,8	-8,0	16,9	1,1
Vĩnh Long	0.94	0.5	2.1	0.2	2.1	-31	40	2.8	2,6	-6,1	16,4	1,1
**Mekong delta**	0.82	-0.5	7.8	0.6	2.6	-82	107	2.8	3,3	-10,7	26,7	1,1

Supplementary Table 2 | List of national benchmarks used for absolute elevation validation. Coordinates are in VN-2000. The national benchmark system in Vietnam is governed by the Department of Survey and Mapping of Vietnam, part of Vietnam's Ministry of Natural Resources and Environment.

National benchmark number	Name of benchmark point	X-coordinate	Y-coordinate	Elevation (m)
657415	Kênh Nam Lộ	627507	1176084	0.660
658439	Thuận Lợi	669678	1158808	0.321
693404	Chắc Tưng	612416	1054971	1.134
693518	Tân Quy A	619041	1063319	-0.285
657423	Kênh Kè	630810	1174045	-0.064
657457	Tấn Đức	663288	1167284	0.375
657417	Ấp 2 Thuỷ Tây	620028	1175109	0.153
681527	Nô Công	658229	1081230	1.262
681535	Sóc Giụp	657528	1077655	0.874
681536	ấp Chợ	661848	1076181	1.120
681529	Chợ Tập Sơn	638001	1076824	1.492
681524	Chòm Chuối	641702	1078533	0.709
681431	ấp Tân Đại	630465	1089604	0.960
681458	Ngãi Hoà	635855	1084992	1.196
669571	Thạnh An	614592	1120402	0.682
669542	An Phú A	611432	1124804	0.858
668526	Kinh Mới	591426	1116280	0.827
668528	Vườn Ba Ngoặc	586585	1114258	1.330
680413	Nhà anh Minh	580295	1100890	0.928
680436	Trường Khánh	582986	1094121	0.977
692401	ấp 8	560201	1068475	0.301
692402	ấp 4	563582	1067544	0.200
704408	Trường Học	574047	1028463	0.768

704417	ấp Kinh Tế Mới	579479	1024004	1.534
703407	Khóm IV	517556	1017633	0.699
703416	Khóm 7	521765	1014021	0.998
679434	Hoà Thạnh	521116	1090743	0.468
679506	Phước Hoà	516503	1103392	0.444
666407	Kinh 1	461989	1139831	0.656
666410	Kinh Hạt	471193	1136886	0.712
643437	Mỹ Phó	517423	1180234	2.259
655413	Mỹ Thiện	521838	1176403	3.515
655450	Bình An II	536672	1159292	2.673
655455	Phú An	533075	1154965	2.229
655499	Phường Mỹ Thới	550364	1144340	1.954
667502	Khóm Thới Thạnh	552205	1140501	1.242
668581	Khóm 4	571832	1137922	1.042
668590	Long Hội	572846	1135765	1.297
655427	ấp 1	546857	1170395	1.711
693512	Trà Tro A	641941	1069227	0.276
667431	Tân Lợi	545830	1125843	0.364
667433	Phụng Thạnh	537481	1124494	-0.534
656454	Ấp 4	578346	1160047	-0.564
656463	Hoà Dân	577710	1158078	-0.381
657452	Bình Tây	658548	1170595	0.122
657453	Bình Đông	659546	1170372	0.234
645440	Hóc Thơm 2	649407	1201527	-0.279
645465	Ấp Thơm 2	648320	1200071	-0.526
681533	Long Trường	646314	1075599	-0.125
681502	Nghĩa Địa	661187	1069833	0.892
655431	Mỹ Phước	517429	1171578	0.212
655475	Vĩnh Qưới	525743	1168909	0.722
657556	Ấp Thạnh Hưng	642161	1149011	-0.223
657447	Ấp 3	639379	1151656	0.403
680576	Phú Thành B	551205	1073548	0.273
692502	Giồng Chùa	547642	1067170	1.236
645416	Kênh Rừng Sến	658489	1204078	1.768
645428	Ấp 3 Nghĩa Trang	642902	1203834	0.295
645431	Hậu Hoà	657106	1203456	2.567
703493	Ấp Thanh Hải	547450	1003072	0.861
704411	Cái Tràm A2-1	571888	1026933	0.239
704409	Hàng Bần	567153	1028257	0.224
703497	Kênh Ba	548363	1001048	0.905
658407	Ấp Phước Hậu Ngoài	675906	1176178	0.49
658414	Ấp Thuận Tây	675337	1169947	-0.237
679442	Hoà Lợi	545476	1085891	-1.172
680523	Ấp Tân Thành	584768	1073450	-0.305
646405	Tân Bửu	586175	1181420	0.153
II-34	Nghĩa Trang	579247	1177640	-0.266

Supplementary Table 3| Elevation statistics for each geomorphological unit based on the Topo DEM and the geomorphological map of Nguyen et al., 2000 (Supplementary Figure 7).

Geomorphological unit	Mean (m)	SD (m)	Area (km^2)
Abandoned channel belt	0.91	0.45	1699
Alluvial apron	1.38	0.77	401
Back swamp	0.66	0.33	1614
Bank: natural levee and crevasse splay	1.38	0.55	1034
Bank: Tidal inlet	0.28	0.16	200
Basement rock	1.20	1.19	24
Channel bar	1.41	0.60	408
Coastal plain	0.53	0.32	7877
Flood basin	0.85	0.46	8205
Flood plain	0.74	0.29	339
Mangrove marsh	0.97	0.48	2123
Marsh	0.34	0.18	2085
Relict beach ridge or sand dune	0.84	0.28	953
Salt marsh	0.83	0.23	1164
Sand spit	1.14	0.20	45
Swamp	0.85	0.50	3006
Tidal Flat	0.99	0.42	151
Undef. dep. of late Pleistocene age	1.84	0.76	511
Weathered land	3.27	1.70	110

Supplementary Table 4| Elevation statistics for each geomorphological unit based on the SRTM elevation model and the geomorphological map of Nguyen et al., 2000 (Supplementary Figure 7).

Geomorphological unit	Mean (m)	SD (m)	Area (km^2)
Abandoned channel belt	2.65	2.38	1699
Alluvial apron	2.84	2.09	401
Back swamp	2.44	3.00	1614
Bank: natural levee and crevasse splay	4.11	3.31	1034
Bank: Tidal inlet	2.29	2.26	200
Basement rock	4.49	4.50	24
Channel bar	3.17	4.11	408
Coastal plain	2.15	2.62	7877
Flood basin	2.83	3.02	8205
Flood plain	2.15	2.41	339
Mangrove marsh	2.35	2.22	2007
Marsh	2.28	2.53	2085
Relict beach ridge or sand dune	2.19	2.83	953
Salt marsh	2.14	2.66	1164
Sand spit	2.67	2.78	35
Swamp	2.57	2.09	3006
Tidal Flat	2.20	2.26	104
Undef. dep. of late Pleistocene age	3.11	1.81	511
Weathered land	6.37	5.19	110

Supplementary Table 5| Elevation statistics for each geomorphological unit based on the MERIT elevation model and the geomorphological map of Nguyen et al., 2000 (Supplementary Figure 7).

Geomorphological unit	Mean (m)	SD (m)	Area (km²)
Abandoned channel belt	3.48	0.96	1699
Alluvial apron	4.19	1.02	401
Back swamp	3.49	0.91	1614
Bank: natural levee and crevasse splay	4.67	1.87	1034
Bank: Tidal inlet	2.68	0.54	200
Basement rock	4.17	4.20	24
Channel bar	5.06	2.39	408
Coastal plain	2.87	0.94	7877
Flood basin	3.54	1.03	8205
Flood plain	3.66	0.57	339
Mangrove marsh	2.29	1.13	2007
Marsh	2.69	0.70	2085
Relict beach ridge or sand dune	2.78	1.24	953
Salt marsh	3.13	1.04	1164
Sand spit	1.58	1.59	35
Swamp	3.73	0.83	3006
Tidal Flat	1.47	1.71	104
Undef. dep. of late Pleistocene age	4.67	0.79	511
Weathered land	6.87	4.77	110

Supplementary Table 6 | Comparison of the validations statistics of different interpolation methods tested to interpolate the topographical elevation points. Interpolation was done using the 3D analyst and geostatistical analysist toolbox in ArcMAP 10.3.1. RMSE = root mean squared error, MD = mean deviation, MAD = mean absolute deviation, std.dev. = standard deviation from mean absolute deviation. A positive deviation means an overprediction of the control point elevation. If cell size is not given, the cell size is 500m x 500m. Only settings which deviate from the default settings are indicated. In all cases, by default the 12 nearest points at a variable search radius are used for interpolation.

Interpolation method	Settings	RMSE (m)	MD (m)	MAD (m)
**Inverse distance weighting (IDW)**	Power: 0.5 Cell size: 1km x 1km	0.50	-0.05	0.25
	Power: 1 Cell size: 1km x 1km	0.50	-0.06	0.25
	Power: 2 Cell size: 1km x 1km	0.51	-0.06	0.25
	Power: 3 Cell size: 1km x 1km	0.53	-0.06	0.25
	Power: 0.5	0.48	-0.05	0.24
	Power: 1	0.48	-0.05	0.24
	Power: 2	0.49	-0.06	0.23
	Power: 3	0.51	-0.06	0.23
**Ordinary Kriging (3D spatial analyst)**	Spherical model	0.53	-0.06	0.24
	Circular model	0.53	-0.06	0.24
	Exponential model	0.53	-0.06	0.24
	Gaussian model	0.47	-0.05	0.24
	Linear model	0.53	-0.06	0.24
**Ordinary Kriging (Geostatistical Wizard)**	Spherical model Number of lags: 20, Lag size: 900m	0.50	-0.05	0.24
	Gaussian model Number of lags: 20, Lag size: 900m	0.50	-0.05	0.24
	Exponential model Number of lags: 20, Lag size: 900m	0.50	-0.05	0.24
**Empirical Bayesian Kriging**	No data transformation, Power model	0.50	-0.04	0.24
	**Empirical data transformation, Exponential model**	0.52	-0.07	**0.22**
**Natural Neighbor**	-	0.52	-0.06	0.25
**Spline**	Tension type spline	0.55	-0.07	0.26
**Topo to Raster (ANUDEM)**	Input type: Point Elevation, No drainage enforcement, Primary type of input data: Spot	0.52	-0.07	0.24
	Input type: Point Elevation, Drainage enforcement, Primary type of input data: Spot	0.53	-0.07	0.24

Supplementary Table 7 | Area and population numbers of the provinces in the Vietnamese Mekong delta (VMD). Total area and provincial population data from the General Statistics Office of Vietnam, available at www.gso.gov.vn. Area Mekong delta plain excludes bedrock, islands and rivers.

**Province**	**Area (km^2)**			**Population in 2016 (persons)**	**Population density in 2016 ($persons/km^2$)**
	**Total area in 2016**	**Main features excluded from total area**	**Mekong delta plain only**		
An Giang	3 537	Bedrock (202 km^2)	3 213	2 159 900	611
Bạc Liêu	2 669	-	2 465	886 200	332
Bến Tre	2 395	Rivers	2 068	1 265 200	528
Cà Mau	5 221	Rivers	5 138	1 222 600	234
Cần Thơ	1 439	-	1 400	1 257 900	874
Đồng Tháp	3 384	Rivers	3 242	1 687 300	499
Hậu Giang	1 622	-	1 622	772 500	476
Kiên Giang	6 349	Islands, bedrock (35 km^2)	5 607	1 776 700	280
Long An	4 495	-	4 458	1 490 600	332
Sóc Trăng	3 312	Rivers	3 148	1 312 500	396
Tiền Giang	2 511	Rivers	2 265	1 740 200	693
Trà Vinh	2 358	Rivers	2 110	1 040 500	441
Vĩnh Long	1 526	Rivers	1 446	1 048 600	687
**Entire VMD**	**40 816**	**Rivers, islands**	**38 181**	**17 660 700**	**433**

Supplementary Discussion

Supplementary Discussion 1

The SRTM and MERIT DEMS and the Topo DEM all contain an elevation anomaly which is likely the result of a measurement or data processing error. In the SW corner of the Ca Mau peninsula, an elevation anomaly is visible in the SRTM DEM (Supplementary Figure 12A) (which translates through to the MERIT DEM). Here, elevation values abruptly change with a sharp, geomorphological unrealistic, transition to several meters below sea level, also clearly visible in the elevation profile (main manuscript Figure 3, profile B-B'). The Topo DEM also contains an elevation inconsistency. In the southern part of the delta along a horizontal line the elevation abruptly changes ~30 cm (Supplementary Figure 12B). This sudden change in elevation is also present in the topographical elevation point dataset on which the Topo DEM is based and may stem from a measurement error or data processing error. It seems to be a local error, as the systematic offset is not present in the eastern part of the delta plain on the same latitude.

Supplementary Methods

Supplementary Methods 1

A range of interpolation methods available in ArcMAP (3D analyst and geostatistical analysist toolbox in ArcMAP V10.3.1.) were used to create different interpolations of the topographical elevation points (Supplementary Table 6). The quality of the interpolated DEMs was tested by a statistical comparison using 120 randomly selected control points (Supplementary Figure 13), which were excluded during the interpolation process. The control points were randomly selected within a squared raster to ensure even distribution over the delta. The DEM interpolated using emperical bayesian kriging with an emperical data transformation and exponential model provided the smallest mean average deviation with the control points (Supplementary Table 6) and contained no visible abnormal elevation artifacts. It was therefore selected as final DEM, presented as Topo DEM in this study. See Supplementary Figure 14 for used settings in Arcmap.

Supplementary References

Kuenzer, C., Guo, H., Huth, J., Leinenkugel, P., Li, X., Dech, S., 2013. Flood mapping and flood dynamics of the mekong delta: ENVISAT-ASAR-WSM based time series analyses. Remote Sens. 5, 687–715. doi:10.3390/rs5020687

Nguyen, V.L., Ta, T.K.O., Tateishi, M., 2000. Late Holocene depositional environments and coastal evolution of the Mekong River Delta, Southern Vietnam. J. Asian Earth Sci. 18, 427–439. doi:10.1016/S1367-9120(99)00076-0
